# Mechanism of Tetrandrine in Ameliorating Hypoxic Pulmonary Hypertension Vascular Remodeling through Transcriptomics and Metabolomics

**DOI:** 10.2174/0113892002393801250812063417

**Published:** 2025-08-28

**Authors:** Xiaowei Gong, Feitian Min, Junli Guo, Ziping Zhang, Xin Liu, Wei Guo, Yaguang Wu, Hanzhou Li, Xixing Fang, Yadong Yuan, Yanling Sheng, Huantian Cui

**Affiliations:** 1 Department of Respiratory and Critical Care Medicine, The Second Hospital of Hebei Medical University, Shijiazhuang 050000, China;; 2 First School of Clinical Medicine, Yunnan University of Chinese Medicine, Kunming 650500, China;; 3 Famous Traditional Chinese Medicine Hall, Tianjin Wuqing District Hospital of Traditional Chinese Medicine, Tianjin 301700, China;; 4 Department of Respiratory and Critical Care Medicine, Hengshui People's Hospital, Hengshui 053000, China;; 5 Department of Respiratory and Critical Care Medicine, Seventh People's Hospital of Hebei Province, Dingzhou 073000, China;; 6 Department of Integrative Chinese and Western Medicine, Tianjin University of Traditional Chinese Medicine, Tianjin 301617, China;; 7 Department of Graduate School, Hubei Minzu University, Enshi 445000, China;; 8 Department of Respiratory and Critical Care Medicine, Huabei Petroleum Administration Bureau General Hospital, Cangzhou 062550, China

**Keywords:** Tetrandrine (TET), hypoxic pulmonary hypertension (HPH), pulmonary vascular remodeling, transcriptomics, metabolomics, arachidonic acid metabolism

## Abstract

**Background:**

Tetrandrine (TET) demonstrates therapeutic potential for hypoxic pulmonary hypertension (HPH); however, its precise pharmacological mechanisms remain unclear. In this study, we aimed to investigate the effects of TET on pulmonary vascular remodeling (PVR) in HPH and elucidate the molecular pathways through which TET ameliorates HPH.

**Methods:**

We established a rat model of HPH and evaluated the therapeutic effects of TET by measuring hemodynamic parameters, assessing right ventricular hypertrophy, and analyzing pathological changes in lung tissue. To explore the molecular mechanisms, we carried out comprehensive analyses using transcriptome and untargeted metabolomics technologies to examine the impact of TET on gene expression and metabolite profiles in the lung tissue of HPH rats. Using data from these multi-omics analyses, we performed biochemical assays, immunofluorescence staining, and Western blotting to validate the effects of TET on vasoconstriction and angiogenesis-related factors. These experiments provide further evidence of the anti-HPH and anti-PVR properties of TET.

**Results:**

TET intervention significantly reduced hemodynamic parameters, including mean pulmonary arterial pressure (mPAP) and right ventricular systolic pressure (RVSP), as well as right ventricular hypertrophy indices, such as the right ventricular hypertrophy index (RVHI) and right ventricle-to-body weight ratio (RV/BW), in HPH rats. TET inhibited smooth muscle cell proliferation and alleviated pathological changes in lung tissue. Transcriptome and metabolome analyses revealed that genes affected by TET intervention were enriched in pathways related to PVR, including those involved in endothelial and smooth muscle cell proliferation, angiogenesis, and blood vessel morphogenesis. Metabolites were predominantly associated with the arachidonic acid (AA) metabolism pathway. Differentially expressed genes included *Cyp4a1*, *Cyp4a3*, *Cyp2u1*, and *Alox15*. Validation experiments demonstrated that TET upregulated ALOX15 protein expression and downregulated CYP4A and CYP2U1 proteins, modulating levels of arachidonate metabolites 20-HETE and 15(S)-HPETE. We further observed that TET reduced the levels of PVR markers, including endothelin-1 (ET-1) secretion, while increasing nitric oxide (NO) release. TET also decreased the expression of cell proliferation markers PCNA and Ki-67 and elevated the endothelial marker CD31. Moreover, TET intervention suppressed angiogenic and vasoconstrictive factors, such as MMP-9, TGF-β1, IGF2, and PDGF-B, while enhancing levels of FGF9 and NOS3.

**Conclusion:**

Our findings highlight the protective effects of TET on lung tissue in HPH mediated through the regulation of 15(S)-HPETE and 20-HETE within the arachidonic acid metabolism pathway. This regulation inhibits pulmonary angiogenesis and vasoconstriction, ultimately improving PVR in HPH.

## 1 INTRODUCTION

Hypoxic pulmonary hypertension (HPH) is a form of pulmonary hypertension (PH) induced by chronic hypoxia and is a significant contributor to global cardiovascular mortality [1]. The pathological mechanisms of HPH include endothelial injury, medial hypertrophy, and adventitial fibrosis in pulmonary vessels. These changes drive pulmonary vascular remodeling (PVR), progressively increasing pulmonary vascular resistance and ultimately leading to right heart failure or death [2]. Despite advances in understanding, the pathogenesis of HPH remains incompletely elucidated due to its complexity. Current treatments for HPH, including oxygen therapy and pharmacological agents, such as prostaglandins and endothelin receptor antagonists, primarily target vasoconstriction. However, these interventions demonstrate limited efficacy in reversing the remodeling of small pulmonary vessels, resulting in suboptimal therapeutic outcomes [3]. This highlights the urgent need to explore the underlying mechanisms of HPH and develop novel therapeutic strategies to slow disease progression.

Hypoxia serves as the primary driver of HPH, inducing metabolic alterations in various lung tissue cells. Non-targeted metabolomics, a high-throughput analytical approach, enables the comprehensive profiling of metabolites under various physiological states, providing valuable insights into disease-specific metabolic mechanisms. Studies using non-targeted metabolomics have consistently linked changes in metabolite levels to the development of HPH. Arachidonic acid metabolism, in particular, plays a critical role in PVR associated with HPH [4]. Disruptions in arachidonic acid metabolism are evident in HPH patients, with arachidonic acid (AA) identified as a potential biomarker for HPH assessment [5, 6]. Research has shown that under hypoxic conditions, lipoxygenases oxidize AA, generating metabolites, such as 15-HETE. These hydrogen peroxide derivatives contribute to dysregulated AA metabolism, exacerbating the pathophysiology of HPH. Abnormal proliferation of vascular endothelial cells (ECs) and smooth muscle cells (SMCs), along with vasoconstriction, plays a critical role in driving PVR [7]. Advances in multi-omics technologies, particularly the integration of metabolomics and transcriptomics, provide powerful tools for investigating the gene expression of enzymes linked to metabolic changes, analyzing biological processes related to these alterations, and uncovering the molecular mechanisms of drug action. Studies have demonstrated that drug therapies can restore arachidonic acid metabolism in HPH models, suppress abnormal SMCs proliferation and migration, and mitigate pathological changes, such as lung wall thickening and PVR [8].

Tetrandrine (TET), a bioactive alkaloid extracted from the roots of *Stephania tetrandra*, has shown the ability to inhibit the proliferation and migration of SMCs, reduce right ventricular hypertrophy, and alleviate vascular hypertension. TET exhibits therapeutic potential for cardiovascular diseases, including HPH [9]. However, its mechanisms for improving PVR in HPH remain inadequately explored. In this study, we first established a hypoxic rat model to evaluate the therapeutic effects of TET on HPH. Using a combination of transcriptomics and non-targeted metabolomics, we analyzed changes in gene expression and metabolite levels in lung tissue following TET intervention. Based on these multi-omics analyses, we investigated the impact of TET on arachidonic acid metabolism. Given the strong association between arachidonic acid metabolism and PVR, we further assessed the ability of TET to ameliorate PVR and examined its role in treating HPH through the lens of metabolic regulation.

## 2 MATERIALS AND METHODS

### 2.1 Animals and Reagents

We used 60 specific-pathogen-free (SPF) grade male Sprague-Dawley (SD) rats, aged 6–8 weeks and weighing 200 ± 10 g, purchased from Beijing Hua Fu Kang Biotechnology Co., Ltd. (Animal License Number: SCXK (Beijing) 2020-0004). The rats were housed in a controlled environment at 20–24 °C with a relative humidity of 50–60% and a 12-hour light/dark cycle. Food and water were available ad libitum. The study was reviewed and approved by the Ethical Review Committee of Animal Experiments in Yunnan University of Chinese Medicine. Moreover, all procedures adhered to the guidelines of animal ethics. Tetrandrine (HY-13764) was obtained from MedChemExpress LLC. Other reagents used in experiments were included in the supplementary materials.

### 2.2 Establishing the HPH Model

After a 7-day acclimatization period, the SD rats were exposed to a low-pressure hypoxic chamber simulating an altitude of 5000 meters, with an oxygen concentration of 10.0 ± 0.2%. The rats underwent continuous hypoxia for 8 hours daily [10, 11]. During this period, we regularly measured mean pulmonary arterial pressure (mPAP) and right ventricular systolic pressure (RVSP). The criteria for establishing a successful HPH model were defined as mPAP ≥ 25 mmHg or RVSP > 40 mmHg [12]. Rats in the control group were maintained under normal environmental conditions. The HPH model was successfully established after 28 days of continuous hypoxia.

### 2.3 Grouping and Drug Intervention

Rats were randomly assigned to six groups using a random number table: Normal (Control), Model (HPH), Sildenafil (SIL), Low-dose Tetrandrine (L-TET), Medium-dose Tetrandrine (M-TET), and High-dose Tetrandrine (H-TET), with 10 rats per group. All groups, except the Control group, underwent the establishment of the HPH model. The SIL group received a daily gavage of 30 mg/kg sildenafil, while the L-TET, M-TET, and H-TET groups were treated with 20 mg/kg, 40 mg/kg, and 80 mg/kg of tetrandrine, respectively. The Control and HPH groups were gavaged with 1.0 mL/100 g of saline daily for 28 consecutive days. Following the model induction and drug treatments, rats were anesthetized with 40 mg/kg pentobarbital sodium. We then measured their mPAP and RVSP. After anesthetizing with pentobarbital sodium, the rats were euthanized by cervical dislocation, weighed (body weight, BW), and their hearts were excised. The right ventricular free wall (RV), left ventricular free wall (LV), and ventricular septum were weighed. Finally, the right ventricle-to-body weight ratio (RV/BW) and right ventricular hypertrophy index (RVHI) were calculated.

### 2.4 Pathological Staining

Lung tissues were collected from rats in each group and fixed in 4% paraformaldehyde solution for 48 hours. After dehydration, the tissues were embedded in paraffin and sectioned into 5-micrometer-thick slices. These sections were stained with Hematoxylin and Eosin (HE) for morphometric analysis. HE staining assessed changes in lung vessel morphology, including the media thickness, external diameter, media cross-sectional area, and total cross-sectional area of pulmonary arterioles. The percentage of media thickness relative to the external diameter of pulmonary arterioles (MT%) and the percentage of media cross-sectional area relative to the total cross-sectional area (MA%) were calculated using pathological image analysis software.

### 2.5 Immunofluorescence

Paraffin-embedded lung tissue sections were dewaxed and rehydrated, followed by incubation in 3% H_2_O_2_ solution for one hour at room temperature. The sections were then blocked for one hour using 20% goat serum. Following PBS washing, the sections were incubated at 4 °C with primary antibodies targeting α-SMA, CD31, PCNA, and Ki-67 for 12 hours. After three washes, the sections were then incubated with fluorescent secondary antibodies for 1 hour (room temperature). The samples were then stained with DAPI (10 mg/mL) and incubated in the dark for 15 minutes. Afterward, we applied an anti-fade mounting medium to the sections and photographed them using a laser confocal microscope. Areas with positive expression were quantified using Image Pro Plus 6.0.

### 2.6 Transcriptomics

Lung tissues were collected before and after TET intervention for transcriptomic analysis, employing methods established in prior research [13]. Specifically, we extracted total RNA from lung tissues in each group and evaluated the purity, concentration, and integrity of the RNA samples. Only samples meeting the quality standards proceeded to library preparation and sequencing on the Illumina platform. Using DESeq2 software, we identified differentially expressed genes (DEGs) by comparing the HPH and Control groups, as well as the TET and HPH groups. DEGs were defined by an FC greater than 1.5 or less than 0.67 and a *p*-value below 0.05. Subsequently, we performed Gene Ontology (GO) enrichment analysis on the identified DEGs.

### 2.7 Metabolomics

Untargeted metabolomics was employed to investigate changes in metabolite levels in lung tissues, following methods documented in the literature [14]. Specifically, we homogenized 100 mg of lung tissues from each group in cold water and centrifuged the samples to obtain the supernatant. Methanol containing internal standards was added, and the mixture was vortexed until homogeneous. We then centrifuged the mixture at 15,000 rpm at 4 °C for 20 minutes, collected the supernatant, and transferred it to UPLC-MS/MS vials. The supernatant was dried using liquid nitrogen, and an external standard solution was added before detection. To ensure consistency, quality control (QC) samples were prepared by mixing equal amounts of supernatant from each group. Using MetaboAnalyst software, differentially abundant metabolites (DAMs) were identified by comparing the HPH group with the Control group and the TET group with the HPH group. DAMs were selected based on an FC greater than 1.5 or less than 0.67, a *p*-value below 0.05, and a VIP score above 1. Finally, Kyoto Encyclopedia of Genes and Genomes (KEGG) enrichment analysis was carried out on the identified DAMs.

### 2.8 Western Blot

Total proteins were extracted from lung tissues, and the concentration of proteins was determined using the BCA method. The extracted proteins were then separated from each group *via* SDS-PAGE electrophoresis. After electrophoresis, we transferred the proteins using the wet transfer method to a PVDF membrane. The membrane was then blocked with a 5% skimmed milk solution for 2 hours. Next, we incubated the membrane with primary antibodies against CYP4A, CYP2U1, ALOX15, MMP-9, TGF-β1, IGF2, PDGF-B, FGF9, and NOS3 overnight at 4 °C. Following three washes with TBST, the membrane was incubated with HRP-labeled secondary antibodies for 2 hours at room temperature. After washing the membrane three additional times, the excess liquid was removed, and the ECL reagent was applied for detection. Using an automatic gel imaging system, we visualized the protein blots. β-Actin served as the internal reference, and the grayscale values of the protein bands were determined using ImageJ.

### 2.9 Biochemical Indicator Detection

ET-1 and NO levels were measured in the serum and lung tissues of rats in each group, using established methods from the literature [15, 16]. To obtain serum samples, blood was collected and then centrifuged at 4000 rpm for 10 minutes. Frozen lung tissue was homogenized with saline at a 1:9 ratio, and the homogenate was centrifuged at 12,000 rpm for 15 minutes to collect the supernatant. We detected ET-1 and NO levels in both serum and lung tissue samples. ET-1 levels were measured using an ELISA kit, while NO levels were quantified by assessing absorbance at 550 nm with a microplate reader. To normalize the tissue samples, the total protein concentration was measured in the homogenates, adhering to the instructions provided with the respective reagent kits.

### 2.10 Statistical Analysis

The normality of the data was assessed using the Shapiro-Wilk test. Normally distributed data were expressed as mean ± standard deviation. For statistical comparisons, a Student's t-test was conducted for two-group analyses and one-way or two-way ANOVA for multiple-group comparisons. A *p*-value < 0.05 was considered statistically significant. All statistical analyses were performed using the SPSS Pro online data analysis platform.

## 3 RESULTS

### 3.1 TET Improved Hypoxia-induced HPH

**Fig. (1):**
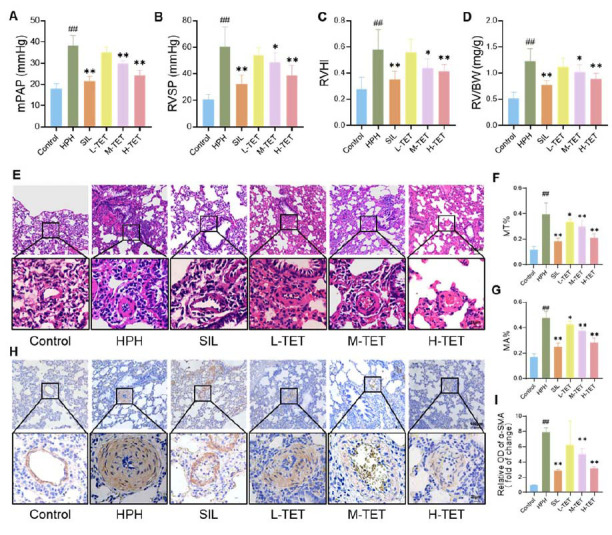
TET improved hypoxia-induced pulmonary hypertension. Rats were placed in a hypoxic chamber simulating high altitude (5000 m above sea level, with an oxygen content of 10.0 ± 0.2%) for 8 uninterrupted hours each day to establish the HPH model. They were simultaneously administered TET at dosages of 20, 40, and 80 mg/kg for 28 days. The therapeutic effects of TET on HPH were evaluated by measuring mPAP, RVSP, RVHI, and RV/BW, and observing histopathological changes in the lung tissue. (**A-D**) TET intervention significantly reduced mPAP (**A**), RVSP (**B**), RVHI (**C**), and RV/BW (**D**) in HPH rats. (**E-G**) HE staining was performed, and the pulmonary arteries in each group were marked using yellow dashed lines (**E**). Quantification of MT% (**F**) and MA% (**G**) was then performed. (H, I) immunohistochemical staining for α-SMA (H) and quantification (**I**). Data are presented as the mean ± SD. n = 10 per group. #*p* < 0.05, ##*p* < 0.01 compared to the Control group; *p < 0.05, **p < 0.01 compared to the HPH group.

After 4 weeks of feeding, rats in the HPH group exhibited significant increases in mPAP, RVSP, RVHI, and RV/BW compared to the Control group. In contrast, rats in the SIL group exhibited normalized mPAP and RVSP, accompanied by a significant reduction in right ventricular hypertrophy. The TET group demonstrated a dose-dependent improvement in hemodynamic parameters and right ventricular hypertrophy (Fig. **1A**-**1D**). HE staining revealed that lung tissue from rats in the HPH group displayed substantial thickening of pulmonary vessels, SMCs proliferation, and luminal asymmetry, along with increased MT% and MA%. In contrast, rats in the SIL group and the L-TET, M-TET, and H-TET groups showed varying degrees of improvement in lung injury (Fig. **1E**-**1G**). Immunofluorescence staining revealed elevated expression of α-SMA in the HPH group, indicating enhanced pulmonary artery muscularization. Both SIL and TET interventions reversed this effect to varying extents (Fig. **1H**-**1I**). Among these, the H-TET and SIL groups showed the most significant therapeutic effects compared to the HPH group, suggesting that TET provides a notable therapeutic benefit in HPH. Based on these results, we selected the high-dose TET for further investigation.

### 3.2 Effects of TET on the Transcriptome of Lung Tissue in HPH Rats

**Fig. (2):**
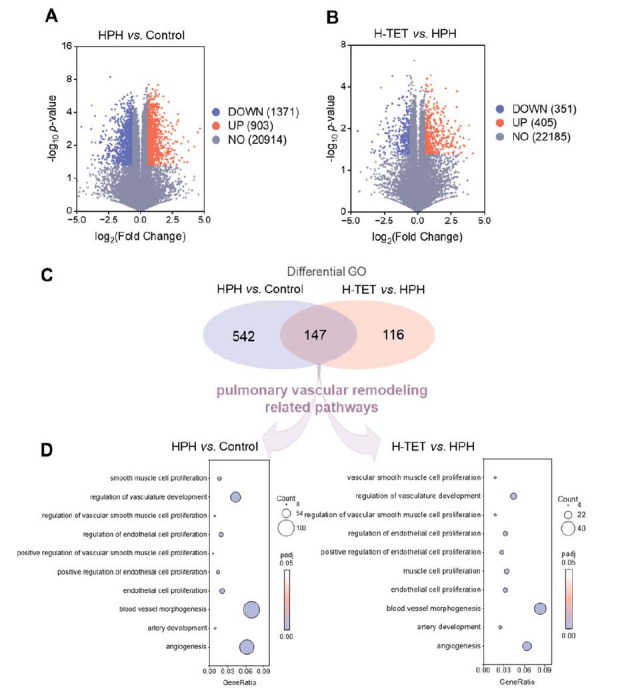
Transcriptome-based evaluation of the impact of TET on differentially expressed genes in HPH rats. We conducted transcriptome analysis on the Control, HPH, and H-TET groups. DEGs between the HPH and Control groups, as well as between the H-TET and HPH groups, were screened using the criteria of FC > 1.5 or < 0.67 and *p-*value < 0.05. (**A**, **B**) Volcano plots used to visualize the DEGs. (**C**, **D**) GO enrichment analysis showing that there were 147 overlapping pathways between the DEGs of HPH *vs*. Control and H-TET *vs*. HPH (C), with significant enrichment in PVR-related pathways, such as smooth muscle cell proliferation (GO:0048659), regulation of vasculature development (GO:1901342), regulation of vascular smooth muscle cell proliferation (GO:1904705), regulation of endothelial cell proliferation (GO:0001936), positive regulation of vascular smooth muscle cell proliferation (GO:1904707), positive regulation of endothelial cell proliferation (GO:0001938), endothelial cell proliferation (GO:0001935), blood vessel morphogenesis (GO:0048514), artery development (GO:0060840), angiogenesis (GO:0001525), muscle cell proliferation (GO:0033002), and vascular smooth muscle cell proliferation (GO:1990874) (D). n = 6 per group.

We performed transcriptome analysis on lung tissue from the Control, HPH, and H-TET groups and identified DEGs using the criteria of a FC > 1.5 or < 0.67 and a *p*-value < 0.05. Fig. (**2A**) shows that, compared to the Control group, 903 genes were upregulated and 1371 genes were downregulated in the HPH group. In comparison to the HPH group, 405 genes were upregulated and 351 genes were downregulated in the H-TET group (Fig. **2B**). Next, we conducted GO enrichment analysis on these DEGs, selecting pathways with a false discovery rate (*p*adj) < 0.05. The analysis revealed 689 enriched pathways in HPH compared to Control and 263 enriched pathways in H-TET compared to HPH. Among these, 147 pathways were common to both comparisons (Fig. **2C**). From these common pathways, we identified those with significant impacts, which were closely associated with ECs proliferation, SMCs proliferation, and angiogenesis. These pathways included endothelial cell proliferation, positive regulation of endothelial cell proliferation, smooth muscle cell proliferation, positive regulation of vascular smooth muscle cell proliferation, angiogenesis, and blood vessel morphogenesis (Fig. **2D**). These pathways play a crucial role in PVR [17]. Our findings suggest that TET may inhibit the PVR process in HPH through these pathways.

### 3.3 Effects of TET on the Metabolome of Lung Tissue in HPH Rats

**Fig. (3):**
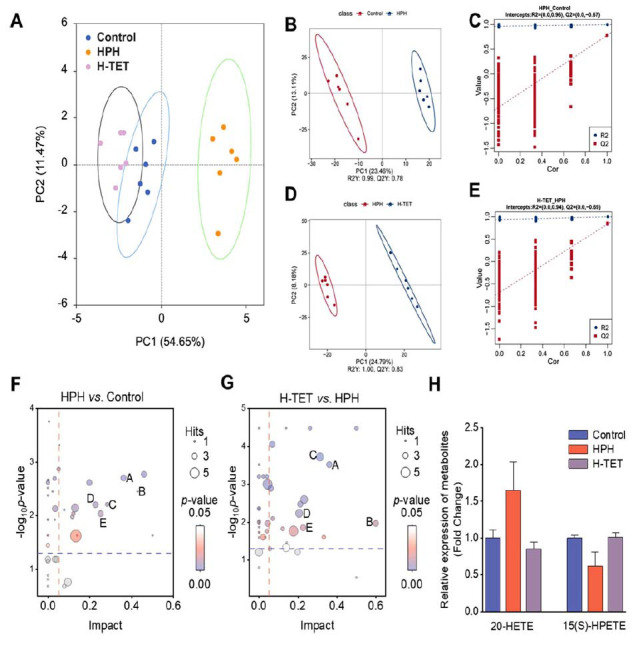
Metabolomics-based evaluation of the impact of TET on differential metabolites in HPH rats. A non-targeted metabolomics analysis was conducted on the Control, HPH, and H-TET groups. (**A**) PCA results showing significant differences in metabolite levels in lung tissues between the Control, HPH, and H-TET groups. (**B-E**) PLS-DA validation results demonstrating good fitting and prediction abilities for both HPH *vs*. Control (**B**, **C**) and H-TET *vs*. HPH (**D**, **E**). (**F**, **G**) KEGG pathway enrichment analysis for HPH *vs*. Control (**F**) and H-TET *vs*. HPH (**G**), where **A**: Arachidonic Acid Metabolism, **B**: Taurine and Hypotaurine Metabolism, **C**: Glutathione Metabolism, **D**: Pyrimidine Metabolism, E: Glycine, Serine, and Threonine Metabolism. (**H**) TET intervention decreased the levels of 20-HETE and increased the levels of 15(S)-HPETE. n = 6 per group.

Principal Component Analysis (PCA) of the untargeted metabolomics data from lung tissue revealed significant differences in metabolite profiles among the Control, HPH, and H-TET groups (Fig. **3A**). We established and validated a predictive model using Partial Least Squares-Discriminant Analysis (PLS-DA). The results showed that for the HPH *vs*. Control comparison, the R^2^ and Q^2^ values ranged from 0 to 0.96 and from 0 to -0.67, respectively. For the H-TET *vs*. HPH comparison, the R^2^ values ranged from 0 to 0.94, while the Q^2^ ranged from 0 to -0.69, suggesting that the model demonstrated both good fitting and predictive power (Fig. **3B**-**3E**).

Next, we identified differential metabolites among the groups using the criteria of a FC > 1.5 or < 0.67, a *p*-value < 0.05, and a VIP score > 1, and performed KEGG pathway enrichment analysis. Key pathway intersections between the HPH and Control, as well as H-TET and HPH comparisons, included glutathione metabolism, glycerophospholipid metabolism, and arachidonic acid metabolism, with arachidonic acid metabolism showing the most significant differences (Fig. **3F** and **3G**). As shown in Fig. (**3H**), compared to the HPH group, the H-TET group showed a significant increase in differential metabolites in the arachidonic acid metabolism pathway, including 15(S)-HPETE, while 20-HETE levels were significantly decreased. Previous studies reported that 15(S)-HPETE exerts anti-angiogenic effects and induces SMCs apoptosis [18, 19], whereas 20-HETE promotes the migration and proliferation of vascular SMCs, exacerbates vasoconstriction, and contributes to pulmonary artery hypertension [20, 21]. Based on these findings, we hypothesize that TET may influence PVR by regulating metabolites in the arachidonic acid metabolism pathway.

### 3.4 Regulation of Arachidonic Acid Metabolism by TET in HPH Rats

**Fig. (4):**
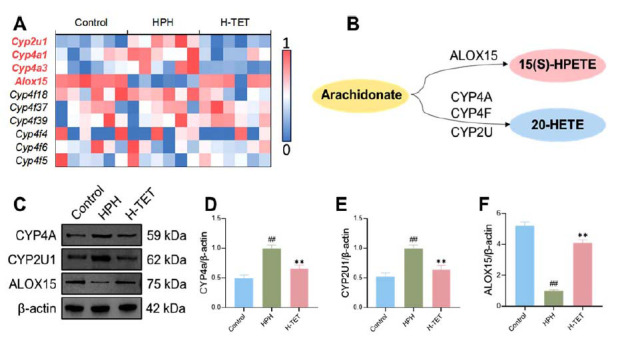
TET intervention regulated arachidonic acid metabolism in HPH rats. Analysis of transcriptome data was conducted to investigate the expression of genes related to arachidonic acid metabolism in lung tissue after TET intervention, further validating the impact of TET on the expression of CYP4A, CYP2U1, and ALOX15 proteins. (**A**) TET intervention downregulated the expression of *Cyp4a1*, *Cyp4a3*, and *Cyp2u1* genes and upregulated the expression of the *Alox15* gene. (**B**) Metabolic relationships among arachidonate, 20-HETE, and 15(S)-HPETE within the arachidonic acid metabolism pathway. (**C-F**) After TET intervention, the expression of CYP4A (**C, D**) and CYP2U1 (**C, E**) was significantly reduced, and the expression of ALOX15 (**C, F**) was significantly increased. n = 6 per group for **A,** n = 3 per group for **C-F**.

Previous studies have demonstrated that in the arachidonic acid metabolism pathway, arachidonate is converted into 20-HETE by the action of CYP4A, CYP4F, and CYP2U enzymes, while it can also be converted into 15(S)-HPETE by ALOX15 [19, 22] (Fig. **4B**). To investigate the mechanism underlying the effects of TET on the arachidonic acid metabolism pathway, we performed a detailed analysis of gene expression changes related to this pathway using transcriptomic data. The results showed that TET intervention downregulated the expression of *Cyp4a1*, *Cyp4a3*, and *Cyp2u1* genes, upregulated the expression of the *Alox15* gene, and had no significant effect on the expression of *Cyp4f4*, *Cyp4f5*, *Cyp4f6*, *Cyp4f18*, *Cyp4f37*, and *Cyp4f39* genes (Fig. **4A**).

Subsequently, we used Western blotting to assess the protein expression levels of CYP4A, CYP2U1, and ALOX15 in lung tissues. The results revealed that, compared to the Control group, protein levels of CYP4A and CYP2U1 were significantly upregulated, while in the HPH group, ALOX15 protein expression was significantly downregulated. TET intervention significantly reduced the CYP4A and CYP2U1 expression and increased ALOX15 expression (Fig. **4C**-**4F**). These findings suggest that TET alleviates HPH by modulating the arachidonic acid metabolism pathway.

### 3.5 TET Improved Pulmonary Vascular Remodeling in HPH Rats

**Fig. (5):**
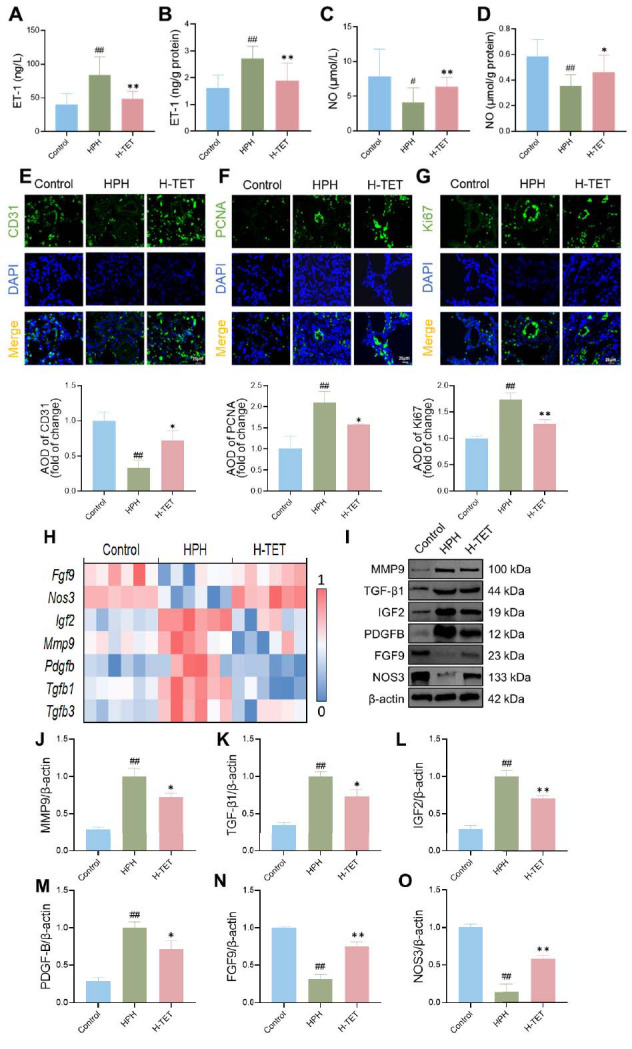
TET intervention improved vascular remodeling in HPH rats. The impacts of TET intervention on ET-1 and NO levels in HPH rats were detected using kits. Simultaneously, immunofluorescence was used to assess the expression of CD31, PCNA, and Ki-67 in the lung tissue. Transcriptome data were analyzed to investigate the differential gene expression related to endothelial cell proliferation, positive regulation of endothelial cell proliferation, smooth muscle cell proliferation, positive regulation of vascular smooth muscle cell proliferation, angiogenesis, and blood vessel morphogenesis after TET intervention. Western blot analysis was performed to validate the effects of TET intervention on the expression levels of MMP-9, TGF-β1, IGF2, PDGF-B, FGF9, and NOS3 proteins. (**A-D**) TET intervention reduced ET-1 (**A**, **B**) and NO (**C**, **D**) secretion in both lung tissue and serum of HPH rats. (**E**-**G**) After TET intervention, CD31 expression increased (**E**), while PCNA (**F**) and Ki-67 (**G**) expression decreased. (**H**) TET intervention downregulated the expression of *Mmp-9*, *Tgf-β3*, *Tgf-β1*, *Igf2*, and *Pdgf-b* genes, and upregulated the expression of *Fgf9* and *Nos3* genes. (**I-O**) Following TET intervention, the protein expression levels of MMP-9 (**I**, **J**), TGF-β1 (**I**, **K**), IGF2 (**I**, **L**), and PDGF-B (**I**, **M**) decreased, while the protein expression levels of FGF9 (I, N) and NOS3 (**I**, **O**) increased. n = 10 per group for A-G, n = 6 per group for H, and n = 3 per group for **I-O**.

Finally, the effects of TET intervention on PVR in lung tissue were assessed. Our analyses of vasoconstriction-related indicators revealed that, compared to the Control group, HPH rats exhibited increased ET-1 secretion and decreased NO release in both lung tissue and serum. TET intervention reversed these alterations (Fig. **5A**-**5D**). Immunofluorescence staining showed a significant increase in the areas positive for PCNA and Ki-67, accompanied by a significant reduction in CD31-positive regions in the lung tissue of HPH rats. After TET intervention, the areas of PCNA and Ki-67 positive regions decreased significantly, while the areas of CD31 positive regions increased significantly (Fig. **5E**-**5G**). We then performed heatmap visualization of differential gene expression within the GO enrichment pathways related to endothelial cell proliferation, positive regulation of endothelial cell proliferation, smooth muscle cell proliferation, positive regulation of vascular smooth muscle cell proliferation, angiogenesis, and blood vessel morphogenesis. The results indicated that TET intervention downregulated the expression of *Mmp-9*, *Tgf-β3*, *Tgf-β1*, *Igf2*, and *Pdgf-b* genes, while upregulating the expression of *Fgf9* and *Nos3* genes (Fig. **5H**). These genes play a critical role in regulating PVR. Western blot analysis further demonstrated that TET intervention reduced the protein expression of MMP-9, TGF-β1, IGF2, and PDGF-B, while increasing the protein expression of FGF9 and NOS3 (Fig. **5I**-**5O**).

## 4 DISCUSSION

Current treatment options for HPH remain limited in efficacy, and the mortality rate among affected patients has not decreased significantly. TET plays a critical role in regulating vasoconstriction and dilation, reversing PVR, and inhibiting SMCs' proliferation in HPH [9]. However, the precise mechanisms by which TET influences HPH treatment remain unclear. In this study, we established a rat model of HPH using a hypobaric hypoxic chamber, a widely accepted method for simulating HPH in research [10, 11]. Hypoxia-induced HPH in rats exhibited key characteristics similar to those observed in clinical patients, including hemodynamic disturbances, right ventricular hypertrophy, and significant thickening of the small pulmonary artery walls. These changes were accompanied by the proliferation of SMCs, asymmetric luminal narrowing, and increased pulmonary vascular muscularization, confirming successful HPH modeling in rats. TET intervention improved hemodynamic parameters and reduced right ventricular hypertrophy in the rats. Pathological analysis showed significant improvements in lung tissue morphology in HPH rats treated with TET. As a positive control, SIL was used, which has been established as an effective treatment for HPH [23]. Our findings demonstrated that high-dose TET was as effective as SIL in ameliorating HPH and reducing PVR, suggesting that TET may serve as a promising therapeutic agent for HPH.

Through combined transcriptome and metabolome analyses, we identified the regulation of AA metabolism as a key mechanism by which TET improves HPH. Our findings demonstrated that TET intervention increases the levels of 15(S)-HPETE while decreasing the levels of 20-HETE metabolites. Additionally, TET downregulates the expression of the *Cyp4a1*, *Cyp4a3*, and *Cyp2u1* genes, while upregulating the expression of the *Alox15* gene. Arachidonic acid metabolism plays a crucial role in regulating vasoconstriction, vasodilation, and cell proliferation and differentiation in HPH. Hence, it represents an important therapeutic target for HPH treatment [24]. AA is a major component of cell membranes, and under hypoxic conditions, it is metabolized in SMCs by the enzymes CYP4A and CYP2U to produce 20-HETE [24, 25]. As a potent vasoconstrictor, 20-HETE contributes to the development of pulmonary vascular hypertension [20, 21, 24]. The binding of 20-HETE to its receptor GPR75 stimulates intracellular Ca^2+^ levels in ECs, leading to endothelial dysfunction [26]. In SMCs, the activation of GPR75 inhibits the activity of calcium- and voltage-activated potassium (BK) channels in SMCs and causes vasoconstriction [27]. Moreover, 20-HETE activates peroxisome proliferator-activated receptor-α (PPARα) to suppress activated protein-1 (AP-1)-mediated COX-2 transcription, promoting the proliferation of SMCs [28]. ALOX15 can also metabolize AA to produce 15(S)-HPETE, a bioactive lipid that inhibits angiogenesis by inducing SMCs apoptosis and plays a key role in regulating PVR [18, 19, 29]. 15(S)-HPETE is reduced to 15-HETE by selenium-containing glutathione peroxidase (GPX), and 15-HETE serves as a key ligand for PPARγ. Their interaction leads to multiple effects: it reduces endothelin-1 (ET-1), increases nitric oxide (NO) release, inhibits the cell survival signaling molecule Akt and the anti-apoptotic protein B-cell lymphoma-2 (Bcl-2), and activates caspase-3. These actions induce apoptosis in endothelial cells (ECs) and contribute to the angiostatic effect [30-33]. Therefore, inhibiting the expression of CYP4A and CYP2U or promoting ALOX15 expression can mitigate PVR. Western blot analysis further confirmed that TET intervention downregulated the expression of CYP4A and CYP2U1 while upregulating the expression of ALOX5. These results suggest that TET alleviates PVR by inhibiting the activity of AA ω-hydroxylases, such as CYP4A and CYP2U1.

Given the strong association between arachidonic acid metabolism and PVR, we investigated the impact of TET on PVR in rats with HPH. Our results demonstrated that TET intervention influenced key PVR-related indicators in HPH rats. Specifically, TET increased NO levels, decreased ET-1 levels, elevated the expression of CD31 and nitric oxide synthase 3 (NOS3), and reduced the expression of proliferating cell nuclear antigen (PCNA) and Ki-67. ET-1, the most potent vasoconstrictor identified to date, is primarily produced by ECs and SMCs. In contrast, NO, produced by NOS3 in ECs, is a vasodilator. The balance between ET-1 and NO is crucial for regulating vascular tone and blood pressure [34, 35]. CD31, a marker of endothelial cells, inhibits endothelial-to-mesenchymal transition (EndMT) and is downregulated in HPH [36, 37]. Meanwhile, PCNA and Ki-67 are markers of cell proliferation that drive SMC proliferation and contribute to PVR in HPH [38, 39]. Additionally, TET intervention modulated the expression of proteins involved in angiogenesis pathways. Specifically, it reduced levels of platelet-derived growth factor-B (PDGF-B), matrix metalloproteinase-9 (MMP-9), transforming growth factor-β1 (TGF-β1), and insulin-like growth factor 2 (IGF2), while increasing fibroblast growth factor 9 (FGF9). PDGF-B is a key factor in vascular cell injury, promoting SMC proliferation, chemotaxis, and migration, and upregulating MMP-9 in ECs [40]. MMP-9, a metalloproteinase that degrades the extracellular matrix (ECM), can activate TGF-β1, which subsequently induces the differentiation of lung myofibroblasts and SMCs proliferation. On the other hand, TGF-β1 can upregulate the expression of MMP-9 [41, 42]. Furthermore, IGF2 promotes angiogenesis by enhancing the recruitment of endothelial progenitor cells and stimulating fibroblast chemotaxis, which are upregulated in patients with HPH and contribute to PH and PVR [43]. In contrast, FGF9, a member of the fibroblast growth factor family, exhibits anti-proliferative, anti-differentiation, and anti-migration effects on SMCs, playing a crucial role in lung tissue repair [44].

## STUDY LIMITATION

Nevertheless, this study did not further conduct cell experiments to verify the specific mecha-nisms underlying the effects of TET intervention on ECs or SMCs. Future studies, incorporating various technologies, such as lipidomics, single-cell sequencing, cellular thermal shift assay (CETSA), and drug affinity responsive target stability (DARTS), can further elucidate the specific targets of TET in improving HPH, laying a solid scientific founda-tion for its development and clinical use.

## CONCLUSION

**Fig. (6):**
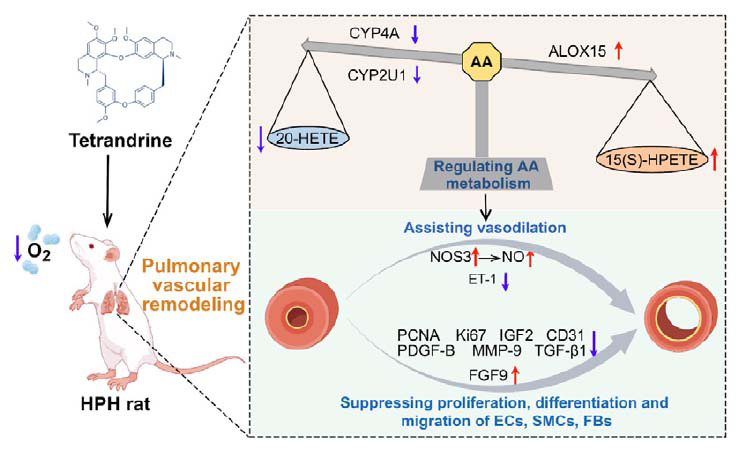
TET inhibits pulmonary angiogenesis *via* regulation of arachidonic acid metabolism, thereby improving vascular remodeling in HPH.

In conclusion, TET shows significant potential as a treatment for HPH. This study, which combines transcriptomics and untargeted metabolomics, has elucidated the mechanism by which TET enhances PVR in HPH, providing a theoretical basis for its clinical application. Specifically, TET regulates key angiogenic processes, including EndMT, SMC proliferation, and migration, by modulating the levels of two metabolites, 15(S)-HPETE and 20-HETE, in arachidonic acid metabolism. These effects improve PVR and protect lung tissue (Fig. **6**). Nevertheless, this study did not further conduct cell experiments to verify the specific mechanisms underlying the effects of TET intervention on ECs or SMCs. Future studies, incorporating various technologies, such as lipidomics, single-cell sequencing, cellular thermal shift assay (CETSA), and drug affinity responsive target stability (DARTS), can further elucidate the specific targets of TET in improving HPH, laying a solid scientific foundation for its development and clinical use.

## Data Availability

The data that supports the findings of the study are available from the corresponding author (Y.Y, Y.S, H.C) upon reasonable request.

## LIST OF ABBREVIATIONS

AA: Arachidonic acid
ECM: Extracellular matrix
ECs: Endothelial cells
EndMT: Endothelial-to-mesenchymal transition
ET-1: Endothelin-1
FGF9: Fibroblast growth factor 9
HPH: Hypoxic pulmonary hypertension
IGF2: Insulin-like growth factor 2
LV: Left ventricular free wall
MMP-9: Matrix metalloproteinase-9
Mpap: Mean pulmonary arterial pressure
NO: Nitric oxide
PCA: Principal Component Analysis
PCNA: Proliferating cell nuclear antigen
PDGF-B: Platelet-derived growth factor-B
PVR: Pulmonary vascular remodeling
RVHI: Right ventricular hypertrophy index
RVSP: Right ventricular systolic pressure
SD: Sprague-Dawley
SMCs: Smooth muscle cells
TET: Tetrandrine
TGF-β1: Transforming growth factor-β1
